# Complex of intratumoral mycobiome and bacteriome predicts the recurrence of laryngeal squamous cell carcinoma

**DOI:** 10.1128/aem.01954-24

**Published:** 2025-02-21

**Authors:** Xinhui Mao, Huiying Huang, Limin Zhao, Feiran Li, Zhenwei Wang, Xiaohui Yuan, Hui-Ching Lau, Chi-Yao Hsueh, Ming Zhang

**Affiliations:** 1 ENT Institute and Department of Otorhinolaryngology, Eye & ENT Hospital, Fudan University 159395 , Shanghai, China; 2 Department of Otorhinolaryngology Head and Neck Surgery, Shanghai Children’s Hospital, School of Medicine, Shanghai Jiao Tong University 56694 , Shanghai, China; Centers for Disease Control and Prevention, Atlanta, Georgia, USA

**Keywords:** fungi, bacteria, laryngeal squamous cell carcinoma, recurrence, alcohol

## Abstract

Dysbiosis of intratumoral fungal and bacterial communities is associated with poor prognosis in various cancers. However, the mycobiome characteristics in laryngeal squamous cell carcinoma (LSCC) and its correlation with recurrence have not yet been investigated. The mycobiome in 80 LSCC samples was characterized using internal transcribed spacer sequencing, encompassing both tumor tissues and their matched para-cancerous tissues. The intratumoral bacteriome was further identified using 16S rRNA sequencing. These two microbial communities were analyzed using bioinformatics and statistical methods to determine its potential correlation with LSCC recurrence. The fungal alpha diversity in tumors was higher compared with that in para-cancerous tissues (*P* < 0.001). A significant difference in the overall fungal community patterns between tumor tissues and para-cancerous tissues was observed based on Bray-Curtis dissimilarity (*P* < 0.001). The presence of *Alloprevotella*, *Porphyromonas*, *Candida*, and *Fusarium* in tumors exhibited a correlation with alcohol consumption. The relative abundance of *Penicillium*, *Exophiala*, and *Aspergillus* in the mycobiome, as well as that of *Alloprevotella*, *Porphyromonas*, and *Peptostreptococcus* in the bacteriome significantly increased the risk of LSCC recurrence (*P* < 0.05). These six microorganisms can combine to form a *microbial complex*, which may independently contribute to recurrence risk in patients with LSCC when enriched within the tumor (hazard ratio = 6.844, *P* < 0.01). Intratumoral fungi and bacteria can be valuable indicators for assessing recurrence in patients with LSCC, indicating their potential as valuable targets for therapeutic intervention.

**IMPORTANCE:**

Our results revealed that dysbiosis of intratumoral microbiota, including increased fungal community diversity and overgrowth of several fungal or bacterial organisms, is substantially linked to the recurrence of LSCC. Drinking habits might alter the laryngeal microbiota to influence the recurrence of LSCC. We also explored a method to potentially predict the recurrence of LSCC from a novel perspective. These findings could offer insights into the etiology of LSCC and pave way to prevent and treat LSCC.

## INTRODUCTION

Laryngeal squamous cell carcinoma (LSCC) is one of the most common pathological types of head and neck cancers (1) and LSCC recurrence often leads to poor prognosis, resulting in numerous deaths each year (2). Therefore, it is crucial to identify predictive markers indicating LSCC recurrence as well as treatment targets.

Studies on the bacterial microenvironment in tumors, including the intestinal bacteriome in colorectal cancer (3) and the oral bacteriome in head and neck cancer (4), are prevalent. Recently, there has been a growing focus on determining the correlation between mycobiome and tumors (5). Pan-cancer analyses indicate the presence of fungi in 35 cancer types, often intracellular, with cell-free fungal DNA showing promise in the diagnosis of early carcinomas (6). Dysbiosis of oral fungal community has been linked to the risk of head and neck cancers (7). Notably, *Candida albicans* has been implicated in promoting the progression of oral squamous cell carcinoma (8). Nevertheless, the fungal community in LSCC has not been explored, and the correlation between the intratumoral mycobiome and LSCC recurrence remains to be studied.

Researches on laryngeal microbiota has been gradually elucidating the mechanisms of LSCC recurrence from novel perspectives. Previous studies have profiled the intratumoral bacterial microbiome of LSCC (9) and revealed that dysbiosis of intratumoral bacterial flora, particularly the enrichment of *Fusobacterium nucleatum*, significantly promotes LSCC progression via various mechanisms (10–13). High levels of *F. nucleatum* are associated with high alcohol consumption and recurrence in patients with LSCC (10). However, to the best of our knowledge, the impact of alcohol consumption on other laryngeal microbiota in patients with LSCC has not been studied.

Therefore, we characterized the fungal composition in LSCC tissues and their adjacent normal epithelial tissues. We also explored the relationship between alcohol consumption and the abundance of intratumoral bacteria and fungi. Furthermore, we determined whether the intratumoral microbiome could serve as an independent risk factor for LSCC recurrence. This study attempted to identify the characteristics of intratumoral microbiota as therapeutic targets and potential prognostic markers for LSCC recurrence.

## MATERIALS AND METHODS

### Patient enrollment and sample collection

A cohort of 80 patients with pathologically confirmed LSCC who underwent surgical treatment at the Department of Otolaryngology at the Eye & ENT Hospital, Fudan University, from October 2017 to November 2019 were enrolled. Exclusion criteria comprised a history of the recent use of antibiotics or antimycotics, active bacterial or fungal infections within the past a month, other malignant diseases, or congenital or acquired immunodeficiency or autoimmune diseases. Detailed information regarding patient demographics, residential and occupational history, family history of malignancies, cigarette smoking habits, alcohol consumption patterns, diabetes mellitus status, and other relevant medical histories was collected using structured electronic questionnaire interviews. Regular telephonic follow-ups were conducted.

To prevent cross-contamination during sample-collection procedures for both tumor tissues and paired para-cancerous tissues, separate surgical instruments were used in a laminar-flow operating room setting. After tumor resection, coffee bean-sized tumor tissue was immediately cut off using a sterile blade and loaded into sterile EP tubes. At the same time, another sterile blade was used to cut normal laryngeal mucosa tissue at least 1 cm from the tumor tissue (14), again the size of coffee beans. The cutting of coffee-bean-sized tissue ensured that the patients’ pathological examination and diagnostic treatment were not compromised, and sufficient DNA was extracted for sequencing study. All collected samples were stored individually at −80℃ for further processing.

### Sample processing and sequencing

Total DNA was extracted following a previously described method (11). Briefly, genomic DNA was extracted from fresh tissues using a QIAGEN DNeasy kit (QIAGEN, Hilden, Germany). To amplify the fungal internal transcribed spacer (ITS) V1 regions, the forward primer (5′-GGAAGTAAAAGTCGTAACAAGG-3′) and reverse primer (5′-GCTGCGTTCTTCATCGATGC-3′) were used on DNA extracted from tumor tissues and para-cancerous tissues. For 16S RNA sequencing of the V3–V4 region, the forward primer (5′-ACTCCTACGGGAGGCAGCA-3′) and reverse primer (5′-GGACTACHVGGGTWTCTAAT-3′) were used on DNA extracted from tumor tissues. The Illumina platform was used for paired-end sequencing of fungal community and bacterial microbiome DNA fragments.

QIIME2 (v. 2019.4.0) software was used for sequence processing. QIIME Cutadapt was used to trim the primer and adaptor sequences (15). DADA2 was used for quality control and denoising (16). Following the acquisition of ASV representative sequences, their length distribution was statistically analyzed using Perl (v. 5.8.3) to ensure comparability with the target fragment length range and to identify the presence of abnormal length sequences. *Nt* database and The BROCC algorithm was used for taxonomic annotation of species both for bacteria and fungi (17, 18). Using the rarefaction method, a specific number of sequences was randomly selected from each sample to achieve a standardized depth (at 95% of the minimum sample sequence size) to predict the observed ASVs along with their relative abundance at the sequencing depth (19).

### Bioinformatics and statistical analyses

Alpha diversity, which refers to the richness, diversity, and evenness of microbial species within a sample or microbial ecosystem, was assessed using Chao1 and observed species for fungal richness, the Shannon and Simpson indices for diversity, and the Pielou index for evenness index. Good’s coverage was used to measure the sample library coverage of the sequencing to reflect whether the sequencing results represented the real situation of the samples. Wilcoxon test was used to compare fungal alpha diversity between tumor tissues and para-cancerous tissues to identify potentially significant predictors of alpha diversity with a *P* value < 0.05.

Beta diversity, which describes the differences in species composition between different communities, was assessed using Principal Coordinates Analysis (PCoA) based on Bray-Curtis dissimilarity. This analysis expanded the sample distance matrix in low-dimensional space while preserving the original distance relationships. Permutational multivariate analysis of variance (PERMANOVA) (Adonis test in “Vegan”) was performed to calculate the variation explained by collected host factors and to determine the significance of the grouping scheme for the variance of the distance matrix, with *P*-values generated from 999 permutations.

To elucidate the differences in mycobiome composition, the species primarily responsible for these differences were analyzed. ASV abundances at the phylum and genus levels for tumor tissues and para-cancerous tissues were calculated and are presented using bar graphs. Wilcoxon test was used to determine the significance of taxonomic differences at various levels. A heatmap was used to visualize the distribution trends of the top 20 fungal genera among samples.

Linear discriminant analysis (LDA) Effect Size (LEfSe) combining the Kruskal–Wallis and Wilcoxon rank-sum tests (20) was used to determine the effect size of the abundance of differential species between groups. A threshold of 4 was set for the promising characterized species. Additionally, a random forest-based machine-learning algorithm was used to construct a classifier model (21), conducting nested hierarchical cross-test analyses to output importance scores for ASVs from all samples to identify the species contributing to differences in fungal community composition between tumor tissues and para-cancerous tissues.

Network analysis was used to identify the patterns of co-occurrence or co-exclusion of microbial communities driven by spatial, temporal, and environmental processes (22). The SparCC algorithm was used to construct the correlation matrix, and the random matrix theory was used to determine the correlation value filtering threshold. The correlation network was constructed using igraph. Node ASVs were divided into peripherals, connectors, module hubs, and network hubs, representing specialists, near-generalists, and super-generalists in the microbial network, respectively (23). The predicted fungal metabolic pathways were analyzed using STAMP, and significant differences between tumor tissues and para-cancerous tissues were identified using Welch’s test.

Receiver operating characteristic curve analysis was used to calculate the optimal cutoff values of the diversity indices and relative abundance of the intratumoral microbes. Kaplan–Meier curves were generated based on the groups defined by the cutoff value, and the log-rank test was used to analyze differences. Cox proportional hazards model was used for univariate and multivariate analyses, with multivariate analysis conducted only if the univariate analysis yielded a significant result (*P* < 0.1). All statistical tests were two-sided and *P* < 0.05 was considered statistically significant.

## RESULTS

### Clinical characteristics of participants and sequencing summary

The clinical information of the 80 patients with LSCC, including age, gender, smoking history, drinking history, subregion, differentiation degree, TNM (tumor, node, and metastasis) stage, and prognosis, is presented in Table 1. At the time of diagnosis, the majority of patients with LSCC were at an advanced stage. Upon follow-up at 76 months, more than half of the patients had not experienced a recurrence.

**TABLE 1:** Clinical characteristics of enrolled patients

Variable	All patients (*N* = 80)
Age at diagnosis, year	
≥60	59 (73.8)
<60	21 (26.2)
Sex	
Male	77 (96.3)
Female	3 (3.7)
Smoking history	
Often	68 (85.0)
Sometimes	4 (5.0)
Never	8 (10.0)
Alcohol history	
Heavy	18 (22.5)
Light	28 (35.0)
Never	34 (42.5)
Subregion	
Supra-glottis	34 (42.5)
Glottis	46 (57.5)
Differentiation degree	
Well	25 (31.3)
Moderate	55 (68.7)
T stage	
Low T-stage (T1–T2)	29 (36.3)
High T-stage (T3–T4)	51 (63.7)
*N* stage	
*N* = 0	53 (66.3)
*N* ≥ 1	27 (33.7)
Overall stage	
Early stage (I–II)	22 (27.5)
Advanced stage (III–IV)	58 (72.5)
Carcinoma recurrence	
Yes	35 (43.8)
No	45 (56.2)
Survival (76 months)	
Yes	51 (63.8)
No	29 (36.2)

In total, 30,336 ASVs of ITS sequencing and 36,277 ASVs of 16S rRNA sequencing were identified from the 80 participants. Prior to taxonomic assignment, ASVs with unclassified taxa annotations (8.4%) of 16S rRNA sequencing were filtered out.

### Comparison of the fungal community diversity in tumor tissues and para-cancerous tissues

**Fig 1:**
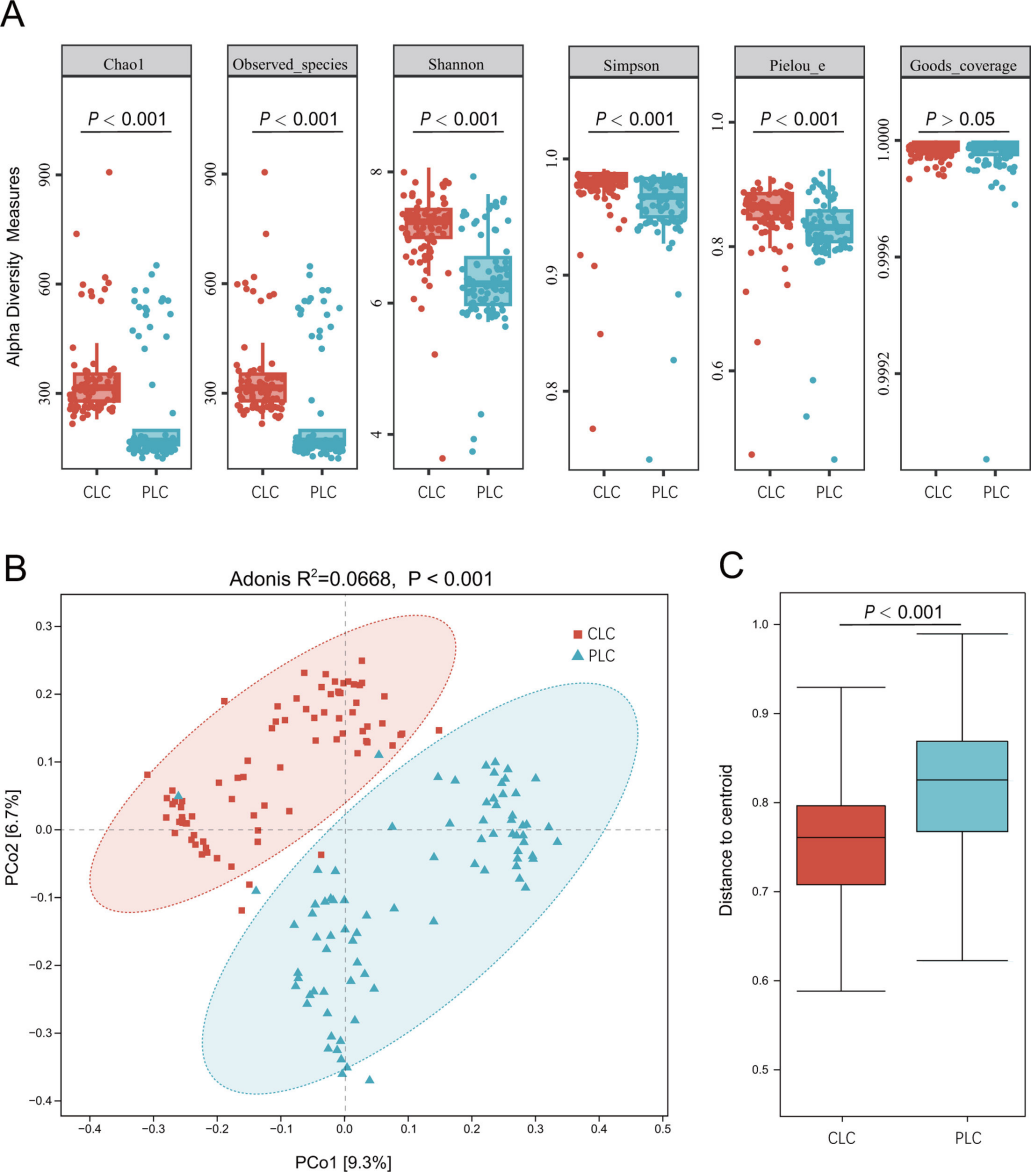
Fungal community diversity analysis in LSCC tissues (CLC) and para-cancerous tissues (PLC). (**A**) CLC harbored significantly higher fungal richness (Chao1 and observed species), diversity (Shannon and Simpson indexes) and evenness (Pielou_e) compared to PLC. Both CLC and PLC have high sample library coverage (Good’s coverage). (**B**) Bray-Curtis dissimilarity-based principal coordinates analysis (PCoA) plot revealed a separation in fungal community structure between CLC and PLC. The axes are labeled with the variation explained, i.e., PC1 explained 9.3% and PC2 explained 6.7% of the variation. (**C**) The average Bray-Curtis dissimilarity in CLC was lower than PLC significantly.

Tumor tissues samples exhibited significantly higher Chao1 index, observed species, Simpson index, Shannon index, and Pielou index compared with para-cancerous tissue samples (Wilcoxon test, *P* < 0.001, Fig. 1A). These findings indicated that LSCC tissues had higher fungal richness, diversity, and evenness than normal epithelial tissues. Both tumor tissues and para-cancerous tissues demonstrated high sample library coverage (Good’s coverage, close to 1.000, *P* = 0.69, Fig. 1A), indicating that the sequencing results accurately represented the samples. Despite some overlap, the PCoA plot showed distinct clustering of fungal communities between tumor tissues and para-cancerous tissues (Adonis test, *P* < 0.001, Fig. 1B and C). In the tumor tissue group, there were no significant differences among the diversity indices mentioned above with respect to factors such as gender (male vs female), age (≤60 years vs older), and smoking status (Fig. S1 to S3).

**Fig 2:**
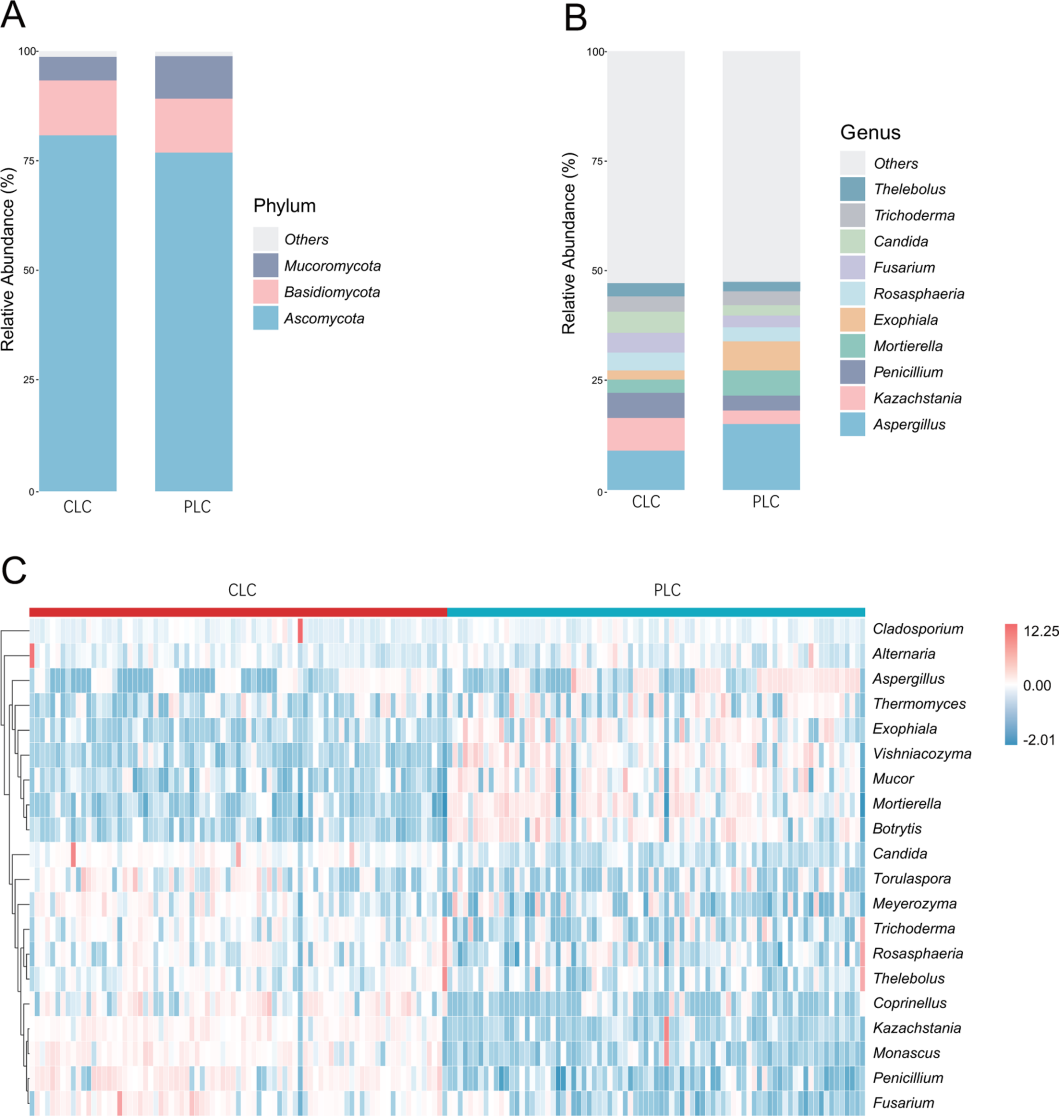
Fungal taxa abundance analysis in LSCC. (**A** and **B**) Present relative abundance of phyla and genera in CLC and PLC. (**C**) The heatmap summarizes the relative abundance of the top 20 abundant genera. The upper-right legend shows the colors that correspond to the relative abundances of genera in each sample. The horizontal axis is arranged according to patient enrollment. The nearest distance method of clustering was used to implement species clustering on the ordinate.

**Fig 3:**
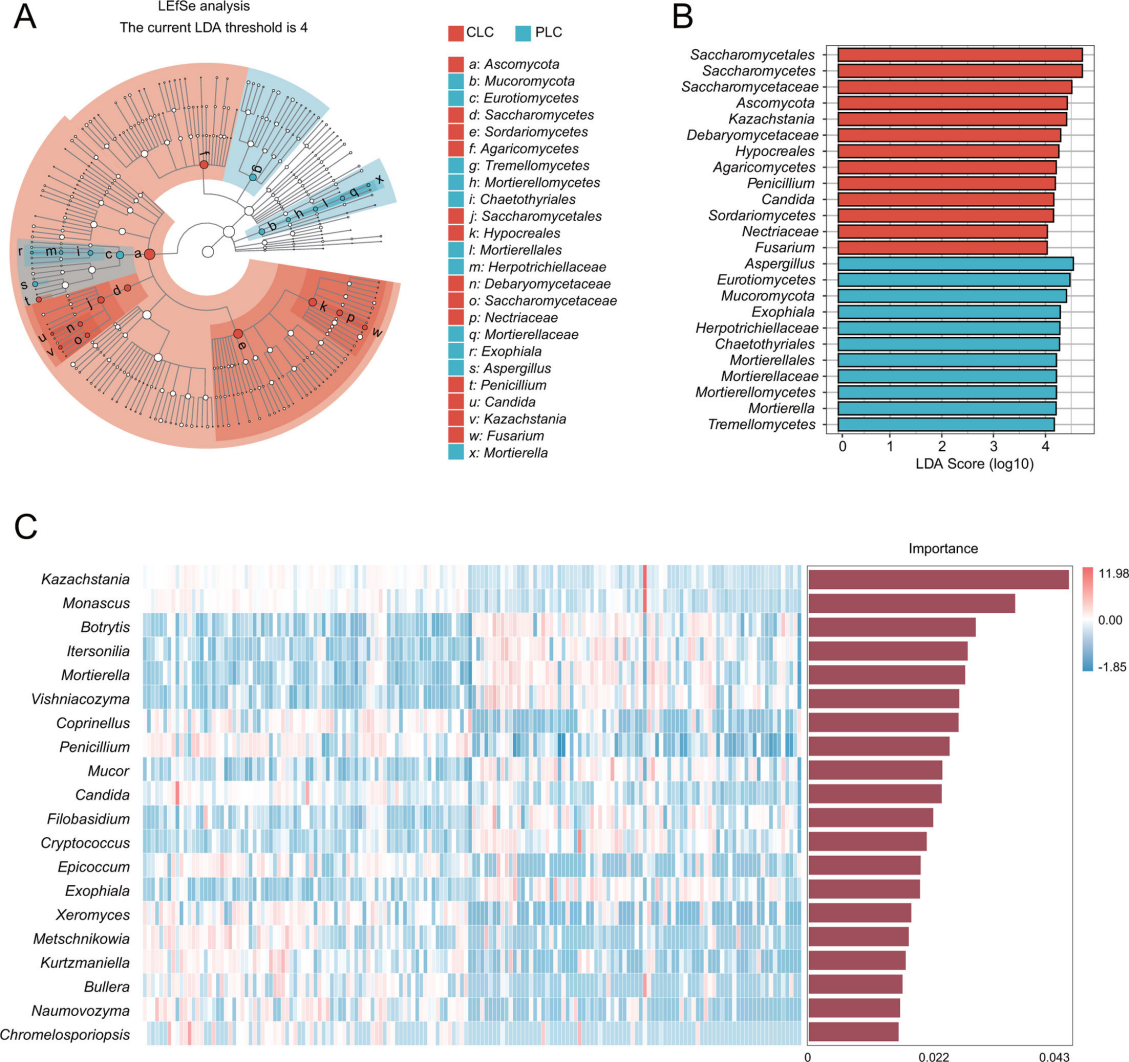
Characterized genera of laryngeal fungal community. (**A** and **B**) Differentially enriched taxa identified by linear discriminant analysis effect size (LEfSe) between CLC and PLC. Cladogram shows the sample composition from phylum to genus classification. Node size corresponds to the mean relative abundance. Nodes in red and blue indicate significant differences between CLC and PLC. The length of the column represents the influence of significantly different species on relative abundance (LDA scores [log10] > 4). (**C**) Random forest analysis for screening the characteristic genera of CLC and PLC. The abscissa is the importance score of the genera to the classifier model, and the ordinate is the taxon name at genus level.

### Analysis of characterized fungal genera and network

The overall fungal community patterns revealed a range of taxa with varying abundances in tumor tissues and para-cancerous tissues. At the phylum level, *Ascomycota*, *Basidiomycota*, and *Mucoromycota* were predominant (Fig. 2A), whereas, at the genus level, *Aspergillus*, *Kazachstania*, *Penicillium*, *Mortierella*, *Exophiala, Rosasphaeria*, *Fusarium*, and *Candida* were dominant (Fig. 2B). A heatmap colored according to the relative abundances shows the predominant genera in the tumor tissues and para-cancerous tissues (Fig. 2C). The difference in relative abundances between tumor tissues and para-cancerous tissues is apparent from the heatmap color distribution.

Using an LDA threshold of 4 for LEfSe analysis, significant differences between tumor tissues and para-cancerous tissues at the genus level were found in *Exophiala*, *Aspergillus*, *Penicillium*, *Candida*, *Kazachstania*, *Fusarium*, and *Mortierella* (Fig. 3A and B). Random forest analysis was used to identify the top 20 genera as potential marker genera, with an overall accuracy of 0.958 (Fig. 3C). Based on the abundance of the genera and statistical analysis, *Exophiala*, *Aspergillus*, and *Mortierella* were suggested as dominant genera in para-cancerous tissues, whereas *Kazachstania*, *Candida*, *Penicillium*, and *Fusarium* were dominant in tumor tissues. The network graph shows the interaction patterns of the top 20 fungal genera (Fig. S4A). While no fungal genus was regarded as a *network hub*, *Exophiala*, *Aspergillus*, *Mortierella*, *Kazachstania*, *Penicillium,* and *Fusarium* which are distributed in the *connector*’s quadrant were close to the *generalists*, indicating important roles in the fungal niche (Fig. S4B). A total of 73 fungal metabolic pathways were found to be significantly different (Welch’s *t*-test, *P* < 0.05, Fig. S5).

### Alcohol consumption and microbiome in LSCC tissues

**Fig 4:**
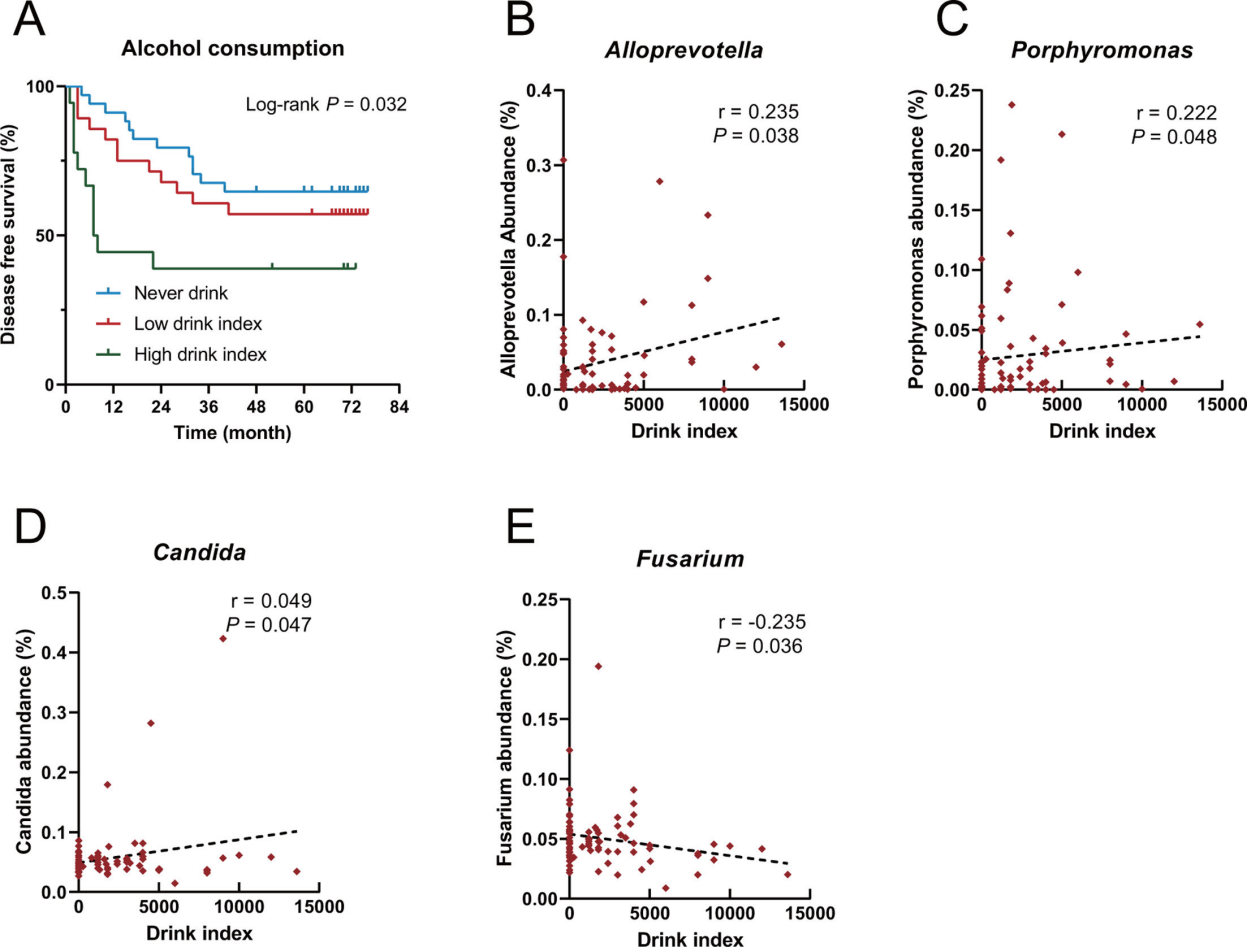
The relationship between alcohol consumption and microbial abundance in LSCC tissues. (**A**) Disease free survival (DFS) of patients with never drink, low and high drink index as assessed by the log-rank test. (**B–E**) The correlation between the abundance of *Alloprevotella*, *Porphyromonas*, *Candida,* and *Fusarium* in LSCC tissues and the drinking index (average alcohol in grams per day multiplied by years of drinking) as assessed with the two-tailed nonparametric Spearman correlation coefficient.

Patients were categorized into never-, light-, and heavy-drinking groups based on a drinking index (average alcohol consumed in grams per day multiplied by years of drinking) threshold of 4,000. Patients with LSCC in the heavy-drinking group had a significantly higher risk of recurrence (log-rank, *P* = 0.032, Fig. 4A). The intratumoral relative abundances of *Porphyromonas*, *Alloprevotella*, and *Candida* were positively associated with the drinking index, whereas the relative abundance of *Fusarium* had a negative association (Spearman rank correlation, *P*＜0.05, Fig. 4B through E).

### Intratumoral microbiome and recurrence of patients with LSCC

**Fig 5:**
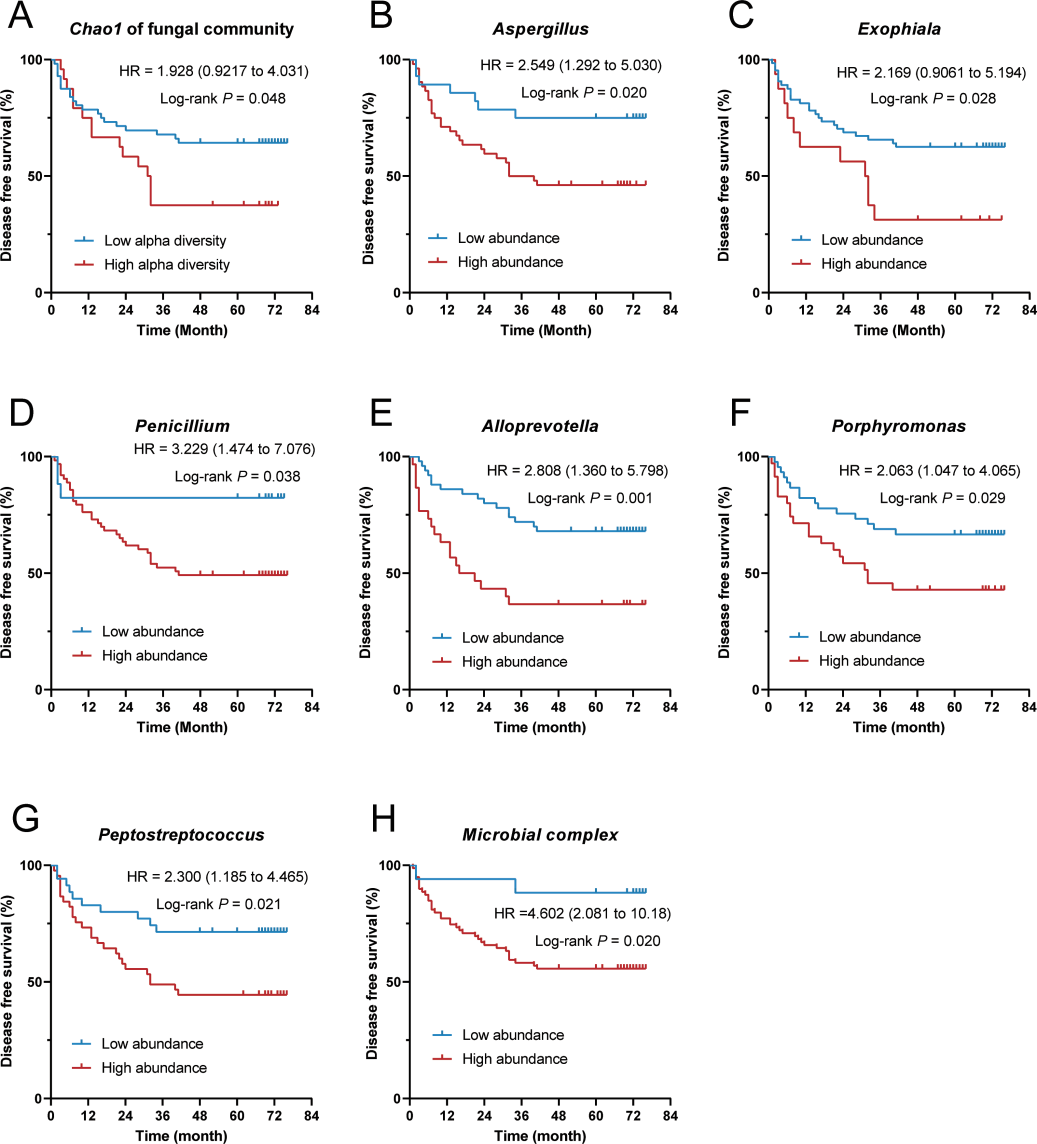
Kaplan-Meier curves for the comparison of intratumoral microbial abundance groups in LSCC patients. (**A**) DFS of LSCC patients with high and low fungal *Chao1* alpha diversity index as assessed by the Log-rank test. (**B–G**) DFS of LSCC patients with high and low abundance of *Alloprevotella*, *Peptostreptococcus*, *Porphyromonas*, *Exophiala*, *Aspergillus,* and *Penicillium* as assessed by the Log-rank test. (**H**) DFS of LSCC patients with high and low abundance of microbial complex (the sum abundance of *Alloprevotella*, *Peptostreptococcus*, *Porphyromonas*, *Exophiala*, *Aspergillus,* and *Penicillium*) as assessed by the Log-rank test.

**Fig 6:**
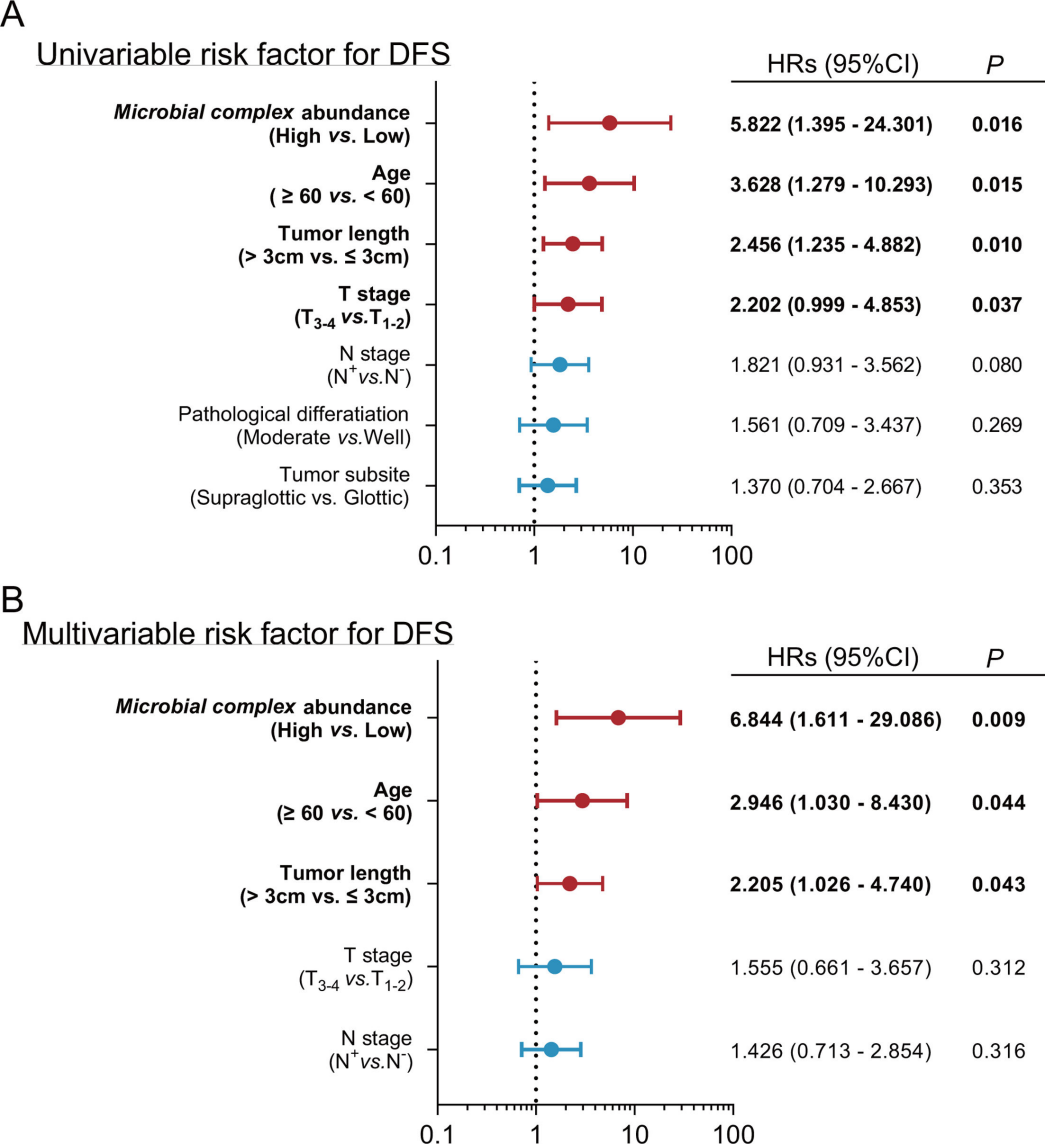
Cox proportional hazards model for possible risk factors of recurrence in LSCC patients. (**A**) Univariate analysis of possible variables for DFS was performed in LSCC patients. The bars correspond to 95% confidence intervals. Data are represented as mean ± SD. (**B**) Multivariate analysis for DFS was performed in LSCC patients. The bars correspond to 95% confidence intervals.

The alpha diversity index (Chao1, *P* = 0.048) of the intratumoral fungal community and the relative abundance of *Penicillium* (*P* = 0.038), *Exophiala* (*P* = 0.028), and *Aspergillus* (*P* = 0.020) were associated with significantly higher recurrence risk (Fig. 5A through D). Among the intratumoral bacterial microbiome, the relative abundances of *Alloprevotella* (*P* = 0.001), *Peptostreptococcus* (*P* = 0.021), and *Porphyromonas* (*P* = 0.029) were significantly linked to a higher risk of recurrence (Fig. 5E through G). The sum of the relative abundances of the above six microorganisms was defined as the microbial complex, which showed a significant correlation with recurrence (*P* = 0.020, Fig. 5H). Univariate analysis showed that age, *T*-stage, tumor length, *N*-stage, and high abundance of microbial complex were significantly related to disease-free survival. Multivariate analysis indicated that the high abundance of microbial complex (hazard ratio = 6.844, 95% confidence interval 1.611–29.086, *P* = 0.009), age ≥60 years, and tumor length >3 cm were independent risk factors of recurrence in patients with LSCC (Fig. 6A and B).

## DISCUSSION

In this single-center retrospective study, LSCC tissues were found to harbor a larger diversity of fungal communities compared with matched para-cancerous tissues. Higher alpha diversity was correlated with an increased risk of LSCC recurrence. Fungal community patterns, measured using beta diversity, also differed significantly between tumor tissues and para-cancerous tissues. Specifically, several fungal taxa exhibited significant differences in abundance between tumor tissues and para-cancerous tissues, including three genera in tumor tissues and four genera in para-cancerous tissues. Moreover, the abundance of certain microbes, including two bacterial genera and two fungal genera, was related to alcohol consumption. Notably, high relative abundances of three bacterial genera and three fungal genera were associated with increased risk of recurrence in patients with LSCC, with high values of the microbial complex acting as an independent risk factor.

Microbial amplicon sequencing is a routine method in tumor microbiology. Specimens can be divided into intra-tumoral and extra-tumoral communities, with the latter including oral fungal communities from saliva (24, 25) or intestinal flora from feces (26). However, the sequencing samples in this study were DNA extracted from LSCC tissues and adjacent normal tissues, emphasizing the intratumoral fungal and bacterial flora interacting with these tissues. As commensal fungi are often present within tumor cells (27, 28), we inferred that the intratumoral microbiota might be a significant effect on LSCC. Previous studies have suggested that poor oral hygiene is a risk factor for head and neck cancer (29–31). Investigating the relationship between oral fungal communities (e.g., saliva or mouthwash) and LSCC could explain the microbiota relationship between the oral cavity and the larynx or pharynx.

We found that the alpha diversity of the intratumoral fungal community, both in terms of richness and evenness, was significantly higher than that in adjacent normal tissues. Intriguingly, higher Chao1 index in tumor tissues showed a higher risk of recurrence. Inconsistent with previous intratumoral mycobiome study in tongue carcinoma (32), high alpha diversity might also be a protective factor in cancers such as head and neck cancer. Furthermore, lower fungal alpha diversity in the oral fungal community was associated with increased odds of nasopharyngeal carcinoma (33), implying that reduced fungal richness might be linked to carcinogenesis. However, owing to the retrospective design of our study, it could not be determined whether the changes in fungal community preceded LSCC diagnosis. Further research should be proceeded to understand whether the variation of fungal community could contribute to LSCC progression. A previous study has suggested that the ecological balance of microbes could promote the immune system to resist pathogens, whereas dysbiosis of commensal microbial communities might lead to immune dysregulation (34, 35). Multiple fungal-immune ecologies were detected across tumors, and intratumoral fungi were found to be spatially associated with cancer cells and macrophages (6). Therefore, it is plausible to speculate a causal relationship between dysbiosis of the laryngeal fungal community and the onset of LSCC.

This study revealed a relationship between the drinking habits and the intratumoral abundance of microbes, including *Alloprevotella*, *Peptostreptococcus*, *Porphyromonas*, and *Fusarium*. Numerous studies have established the association between alcohol consumption and the recurrence of LSCC (36, 37). Alcohol may play a role in tumor progression by affecting the composition of the laryngeal microbiota (38). The positive feed-forward loop between *F. nucleatum* and alcohol metabolism reprogramming affects the prognosis of patients with LSCC (10). Alcohol could modulate *Candida albicans* to induce carcinogenesis and progression of oral cancer *in vitro* (39). Drinking alters the abundance of the oral microbiota, including that of *Alloprevotella*, modulating the progression of alcohol-related liver disease (40). *Porphyromonas* in gut microbiota is associated with voluntary alcohol consumption (41). Therefore, we hypothesized that drinking may also alter the laryngeal microbiota to influence the recurrence of LSCC, necessitating further validation with a larger sample size as well as mechanistic investigation.

The high relative abundance of *Penicillium*, *Exophiala*, *Aspergillus*, *Alloprevotella*, *Porphyromonas*, and *Peptostreptococcus* were identified as risk factors for recurrence in patients with LSCC. The Cox proportional hazards model, along with the high microbial complex comprising the above six microorganisms, could be regarded as an independent risk factor of recurrence in patients with LSCC. One possible mechanism for the increased microbial abundance could be that patients were immunocompromised before LSCC onset, contributing to the overgrowth of pathogenic or opportunistic pathogenic microbiota (42).

Many studies in recent years have shown that microbiota plays an important role in cancer treatment. Gut microbiome serve as not only biomarkers but also potential targets for enhancing the efficacy of immunotherapy (43), while their metabolites could predict adverse events in neoadjuvant therapy for rectal cancer (44). Prodigiosin, a metabolite of *Serratia*, showing significant inhibiting effect of a variety of cancers and deemed as potential drug for cancer therapy (45). Modulation of gut microbiota by probiotics could improve the efficacy of immunotherapy in hepatocellular carcinoma (46). Probiotic-nanomedicine conjugate biohybrid have been designed for enhanced cancer chemo-immunotherapy (47). In future studies, probiotics might improve the prognosis of LSCC patients by regulating microbial complex in LSCC. Different from gut microbiota, the study of laryngopharyngeal microbiota is still limited and we look forward to more studies to clarify the potential role of laryngeal microbiota in the treatment of LSCC.

Some limitations in our study should be acknowledged. First, we could not establish a temporal and causal relationship between dysbiosis of the intratumoral mycobiome and the recurrence risk of LSCC due to the retrospective nature of this study. Second, given the limited sample size at a single center, it is uncertain whether our findings can be generalized to a broader population. Lastly, due to the limitations of ITS and 16S sequencing methods, taxa classification is reliable only at the genus level, and conclusions at the species or subspecies level will depend on advanced sequencing technology (48).

Overall, our results revealed that dysbiosis in the intratumoral microbiota, including increased mycobiome diversity and overgrowth of several fungal or bacterial organisms, is substantially linked to the recurrence of LSCC. Drinking habits might alter the laryngeal microbiota to influence the recurrence of LSCC. Combined mycobiome and bacteriome could potentially predict the recurrence of LSCC. These findings, if confirmed based on future multicenter prospective studies, could offer insights into the etiology of LSCC and pave way to prevent and treat LSCC.

## Data Availability

The raw sequence data reported in this paper have been deposited in the Genome Sequence Archive of the National Genomics Data Center, China National Center for Bioinformation/Beijing Institute of Genomics, Chinese Academy of Sciences, and are publicly accessible under accession no. HRA009600.

## SUPPLEMENTAL MATERIAL

Supplemental figures aem.01954-24-s0001.docx Figures S1 to S5.

ASM does not own the copyrights to Supplemental Material that may be linked to, or accessed through, an article. The authors have granted ASM a non-exclusive, world-wide license to publish the Supplemental Material files. Please contact the corresponding author directly for reuse.
