# Supplementary material for: Complex of intratumoral mycobiome and bacteriome predicts the recurrence of laryngeal squamous cell carcinoma
Source: Appl Environ Microbiol. 2025 Feb 21;91(3):e01954-24. doi: 10.1128/aem.01954-24 (PMC11921384; doi:10.1128/aem.01954-24)
Supplement: Supplemental figures — Figures S1 to S5. [file aem.01954-24-s0001.docx]

**Supplemental Material**

**Complex of Intratumoral Mycobiome and Bacteriome Predicts the Recurrence of Laryngeal Squamous Cell Carcinoma**

**Xin-hui Mao^1, 3^, Hui-ying Huang^1,^** **^3^, Limin Zhao^2,^** **^3^, Fei-ran Li^1^, Zhen-wei Wang^1^, Xiao-hui Yuan^1^, Hui-Ching Lau^1^, Chi-Yao Hsueh^1,^ *, Ming Zhang^1,^ ***

^1^ ENT institute and Department of Otorhinolaryngology, Eye & ENT Hospital, Fudan University, Shanghai, 200031, China

^2^ Department of Otorhinolaryngology Head and Neck Surgery, Shanghai Children’s Hospital, School of medicine, Shanghai Jiao Tong University

^3^ These authors contributed equally to this work: Xin-hui Mao, Hui-ying Huang and Limin Zhao

*Corresponding authors:

Ming Zhang, (Email: ent_zhm@126.com)

Chi-Yao Hsueh, (Email: hsuehchiyao@gmail.com)


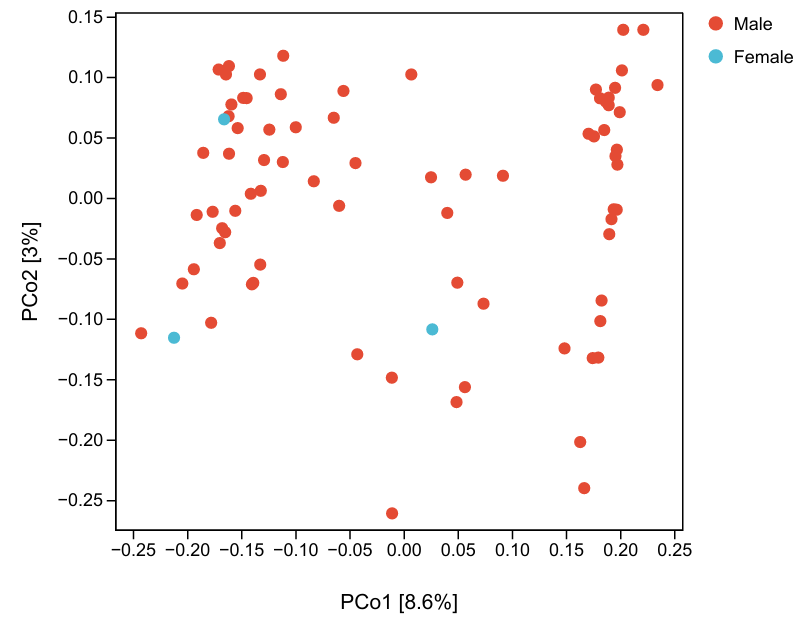

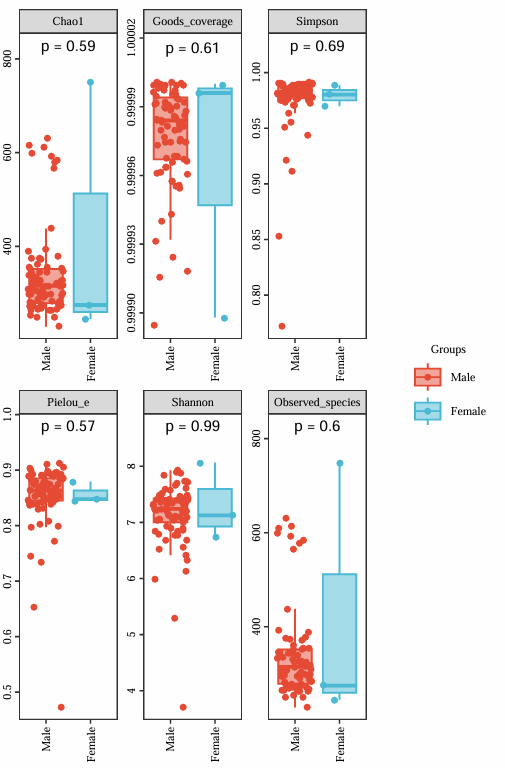


**Figure S1.** There were no significant differences among the diversity indices mentioned above with respect to gender (male or female).


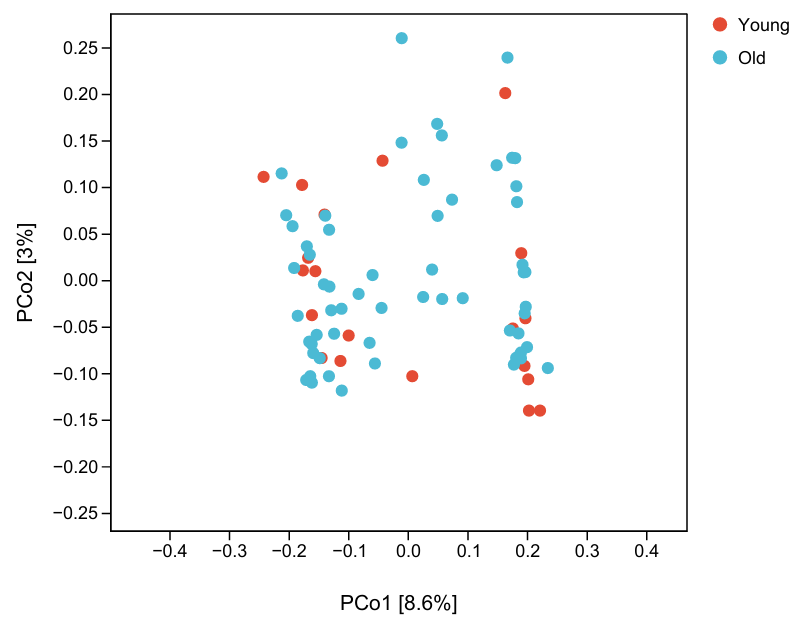

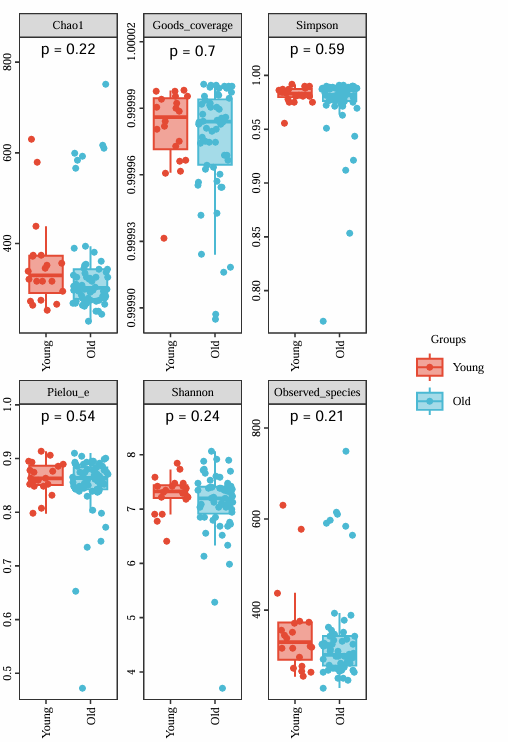


**Figure S2.** There were no significant differences among the diversity indices mentioned above with respect to age (≤ 60 years vs. older).

**
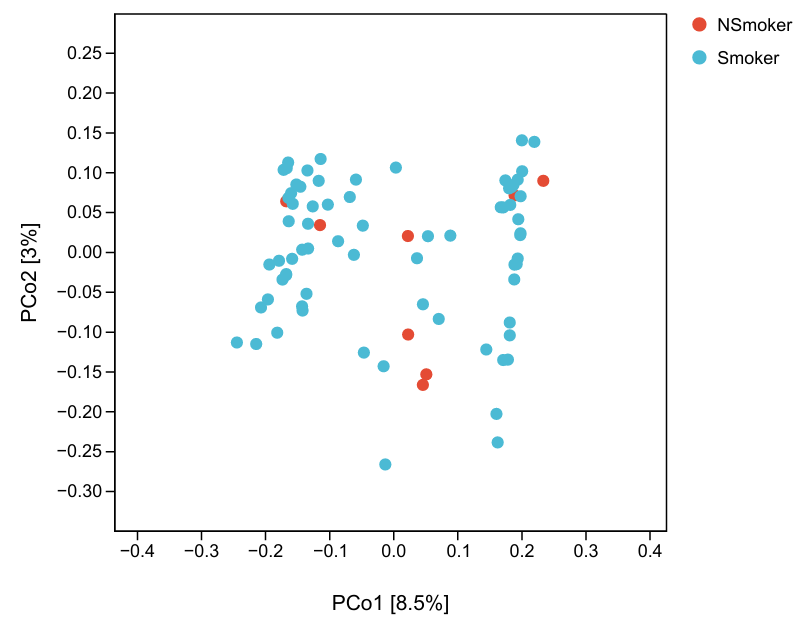

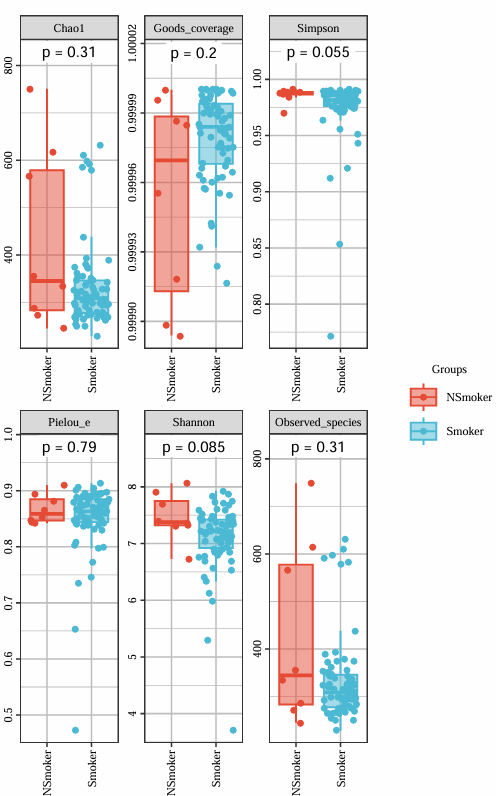
**

**Figure S3.** There were no significant differences among the diversity indices mentioned above with respect to smoking status (Not/sometimes smoker or often smoker).


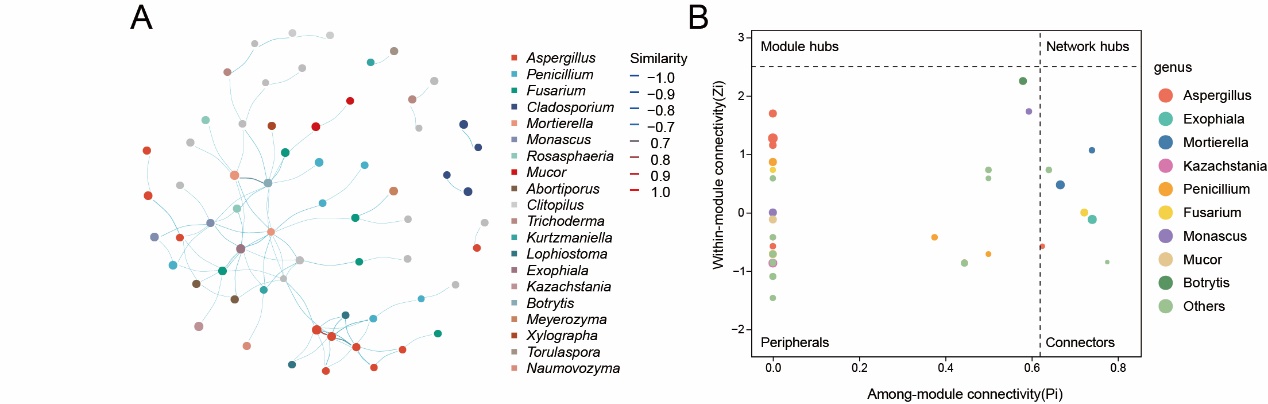


**Figure S4. Network analysis of LSCC fungal community**

(A) Fungal genera network of LSCC patients. Each node representatives a fungal genus. The size of the node is positively relative to its abundance. The edge between nodes indicates the correlation and the color of the edge represents the similarity.

(B) Zi and Pi values divide the node ASV in the network into peripherals, connectors, module hubs and network hubs. The fungal genera which distributed in the connector’s quadrant were close to generalists.


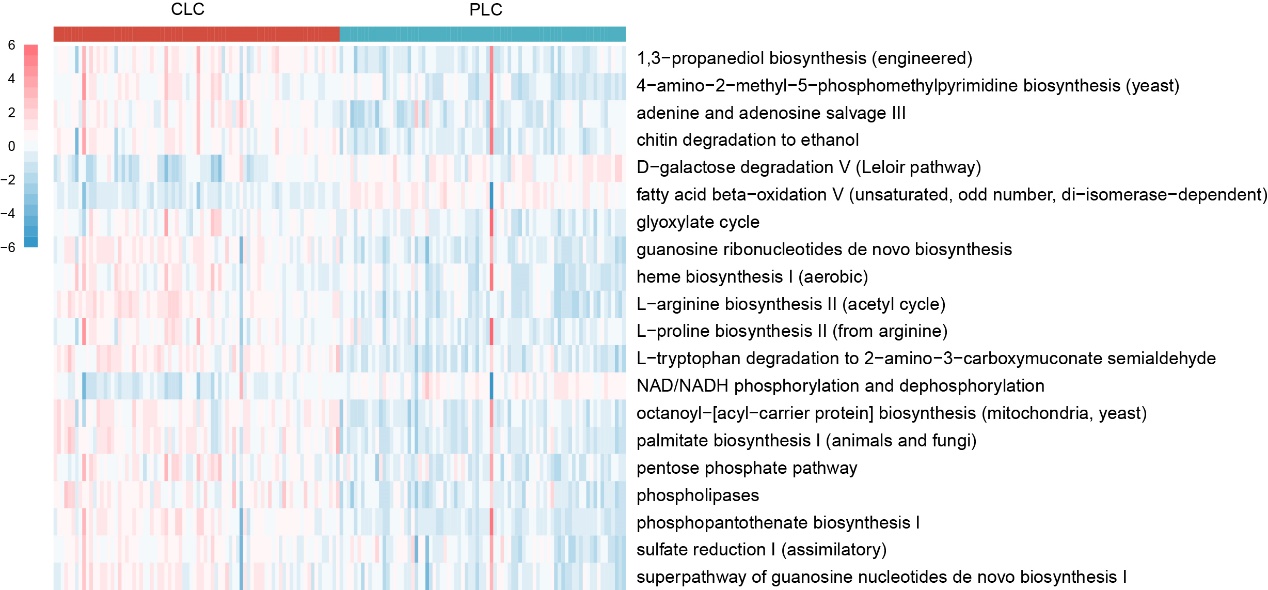


***Figure* S5: Metabolic Pathway Differences in LSCC tissues and normal tissues.**

The heatmap depicts the major metabolic pathway between groups. The upper-right legend shows the colors that correspond to the relative abundances of pathway in each sample. The horizontal axis is arranged according to patient enrollment.
